# Inflammatory microenvironment in the initiation and progression of bladder cancer

**DOI:** 10.18632/oncotarget.21565

**Published:** 2017-10-06

**Authors:** Xinbing Sui, Liming Lei, Liuxi Chen, Tian Xie, Xue Li

**Affiliations:** ^1^ Department of Medical Oncology Holistic Integrative Oncology Institutes and Holistic Integrative Pharmacy Institutes, The Affiliated Hospital of Hangzhou Normal University, College of Medicine, Hangzhou Normal University, Hangzhou, China; ^2^ Department of Medical Oncology Holistic Integrative Cancer Center of Traditional Chinese and Western Medicine, The Affiliated Hospital of Hangzhou Normal University, Hangzhou, China; ^3^ Departments of Urology and Pathology, Boston Children's Hospital, Boston, MA, USA; ^4^ Department of Surgery, Harvard Medical School, Boston, MA, USA; ^5^ Department of Cardiovascular Surgery of Guangdong Cardiovascular Institute, Guangdong General Hospital, Guangdong Academy of Medical Sciences, Laboratory of South China Structural Heart Disease, Guangzhou, China; ^6^ Department of Medical Oncology, Sir Run Run Shaw Hospital, College of Medicine, Zhejiang University, Hangzhou, China

**Keywords:** inflammation, tumorigenesis, development, bladder cancer

## Abstract

Accumulating evidence suggests the idea that chronic inflammation may play a critical role in various malignancies including bladder cancer and long-term treatment with non-steroidal anti-inflammatory drugs (NSAIDs) is significantly effective in reducing certain cancer incidence and mortality. However, the molecular mechanisms leading to malignant transformation and the progression of bladder cancer in a chronically inflammatory environment remain largely unknown. In this review, we will describe the role of inflammation in the formation and development of bladder cancer and summarize the possible molecular mechanisms by which chronic inflammation regulates cell immune response, proliferation and metastasis. Understanding the novel function orchestrating inflammation and bladder cancer will hopefully provide us insights into their future clinical significance in preventing bladder carcinogenesis and progression.

## INTRODUCTION

Chronic inflammation has been recognized as one of the hallmarks of cancer. In early 1989, Rudolf Virchow hypothesized that cancer originated from the sites of chronic inflammation [1]. Since then, a large amount of studies investigated the association between the inflammatory microenvironment of malignant tissues and cancer prevention or treatment, and accumulating evidence has supported Virchow's hypothesis [2, 3]. Inflammation is an essential host defense mechanism for cell or organism injury in response to stresses, by which the immune system tries to neutralize or eliminate injurious stimuli and initiate regenerative or healing processes. However, excessive or persistent inflammation is also shown to contribute to carcinogenesis and tumor progression by activating a series of inflammatory molecules and signals [4, 5].

Bladder cancer (BCa) is the fourth most common cancer among men and the ninth overall in the world [6]. Males are more likely to develop BCa than females. When diagnosed, 90% of bladder cancers are urothelial carcinomas, whereas the remaining 10% are mostly squamous cell carcinoma (SCC) and adenocarcinomas [7]. Many risk factors contribute to the malignant transformation and the progression of bladder cancer, including smoking, heavy alcohol consumption and occupational exposure to polycyclic aromatic hydrocarbons or aromatic amines [8, 9]. Besides these risk factors, chronic inflammation is recently recognized as another risk factor for BCa [10].

Recent studies have linked inflammation with the formation and development of bladder cancer. On the one hand, chronic inflammation, whether systemic or local, increases the risk of developing BCa. On the other, the oncogenic changes may induce a chronically inflammatory microenvironment which has many tumor-promoting effects on the cell proliferation, angiogenesis, invasion and metastasis of bladder cancer [11, 12]. However, the molecular pathways involved in inflammation-related bladder cancer still remain largely unexplained. Thus, there is an urgent need to understand the underlying molecular mechanisms on the possible role of chronic inflammation during bladder carcinogenesis. In this review we will summarize the different cellular and molecular signaling pathways regarding the relationship between chronic inflammation and bladder cancer promotion and progression, and discuss the attractive prospect of targeting inflammation as a revolutionary strategy for cancer prevention and therapy.

## THE TRIGGERS OF CHRONIC BLADDER INFLAMMATION

### Urinary tract infection

Urinary tract infections (UTIs) are among the most common urologic diseases. About 80% of UTIs occur in women and 40–50% of females have at least one symptomatic infection during their lifetime [13]. The majority of UTIs are caused by *Escherichia coli* (80%) or *Staphylococcus saprophyticus* (10–15%) [14]. Previous epidemiological studies have reported a correlation between UTI and the increased risk of BCa. The majority of these studies show UTI not only increases the risk of BCa but also is associated with worse BCa outcomes, in both men and women [15–17]. However, there is a conflicting report regarding the potential role of UTI. Jiang *et al*. reported that a history of UTI was associated with a reduced risk of BCa among women [18]. Possible mechanisms that were involved in this paradox might be the cytotoxicity against BCa cells from the antibiotics commonly used to treat bladder infections. Recently, Vermeulen *et al*. investigated the association between UTI and the risk of BCa, and they found that regular cystitis was positively associated with the risk of BCa, whereas, those women with episodes of UTI treated with antibiotics have a decreased urinary bladder cancer (UBC) risk [19].

*Schistosoma haematobium* (*S. haematobium*), endemic in Africa and the Middle East, is a chronic infection caused by parasitic *Schistosoma* worms. There is strong evidence linking *S. haematobium* infection with increased bladder cancer incidence [20, 21]. In fact, *S. haematobium* is particularly relevant with squamous cell carcinoma of the bladder [22]. *S. haematobium* antigens have been observed to induce the development of urothelial dysplasia and inflammation [21]. In the experiment, *S. haematobium* was shown to have carcinogenic ability through enhanced c-KIT expression or oncogenic mutation of KRAS gene [23, 24]. The nuclear localization of cyclooxygenase-2 (COX-2) was also involved in *S. haematobium*-mediated stem cell differentiation/proliferation in bladder carcinogenesis through upregulation of Oct3/4 expression [25]. In addition, p53 signaling seems to mediate urothelial malignant transformation during *S. haematobium* infection in a sex-specific manner, but it's not clear if p53 actually inhibits urothelial cell cycle progress and carcinogenesis in the setting of urogenital *schistosomiasis* [26, 27].

### Human papilloma virus (HPV)

Human papillomavirus (HPV) infection has been known as a risk factor for certain cancers such as cervical, anogenital, oropharyngeal carcinoma and skin cancers [28–30]. However, whether the virus might play a key role in the pathogenesis of BCa has not been well clarified. A number of studies have been done to elucidate this possibility. The meta-analysis from Li *et al*. reported that HPV infection were significantly associated with the increased risk of BCa [31]. However, a modified meta-analysis suggested limited biologic rationale for a role of HPV in BCa [32]. Kim *et al*. provided some evidence that HPV infection may be associated with squamous metaplasia of the bladder especially in non-smokers [33]. The work from Shigehara *et al*. showed that HPV may play an etiological role in the tumorigenesis of female BCa at younger patients with a past history of cervical cancer, however, no statistical difference was observed between high-risk HPV infection and histologic subtypes of BCa [34]. Currently, the molecular subtypes of BCa have been identified and HPV infection may have a role in the development of a small percentage of urothelial carcinoma patients with amplified and overexpressed BCL2L1 [35]. In summary, although a large number of research that has shown the presence of HPV in BCa, the results are far from conclusive.

### Chronic chemical and mechanical irritations

It has been suggested that some chronic irritations of urinary tract are also risk factors for BCa. When bladder mucosa is persistently subjected to these chronic irritations, the urothelium tend to generate a high level of cell proliferation, resulting in many pathological changes such as dysplasia, metaplasia, carcinoma *in situ* and ultimately to invasive carcinoma [36, 37]. N-butyl-N-(4-hydroxybutyl) nitrosamine (BBN), an alkylating agent, is the most commonly-used chemical inducer of murine BCa model [38]. The uracil, a nongenotoxic chemical, can induce urinary bladder carcinomas in rats and mice, which was related to the presence of calculi in the urinary bladder and increased spontaneous mutations by vigorous cell proliferation [37, 39]. Long-standing bladder stones have been also implicated as a cause of urinary tract cancers [40, 41], however, the association between urinary stones and BCa is largely undefined. In addition, some foreign bodies such as pellets of paraffin wax, glass beads and wood chips were demonstrated to induce urothelial tumorigenesis [42–44]. Chronic indwelling urinary catheters (CIDCs) and augmentation cystoplasty are also considered as risk factors of BCa development, especially in older aged and male patients [45]. Augmentation cystoplasty is the gold standard treatment for the patients with congenital bladder abnormalities. The question is whether these patients have an increased risk of BCa. A number of early studies showed that the patients with surgical bladder augmentation had an increased risk of BCa [46, 47]. However, there are also conflicting reports regarding an increased risk of malignancy after augmentation cystoplasty. Higuchi, *et al*. explored the relationship between augmentation and cancer, and they found that augmentation did not appear to be an independent risk factor for the development of BCa [48, 49]. In order to clearly understand the relationship between augmentation cystoplasty and the formation of BCa, large-scale and multicenter collaboration will be necessary in the future.

## INFLAMMATORY CELLS AND CYTOKINES IN TUMOR MICROENVIRONMENT OF BLADDER CANCER

**Figure 1:**
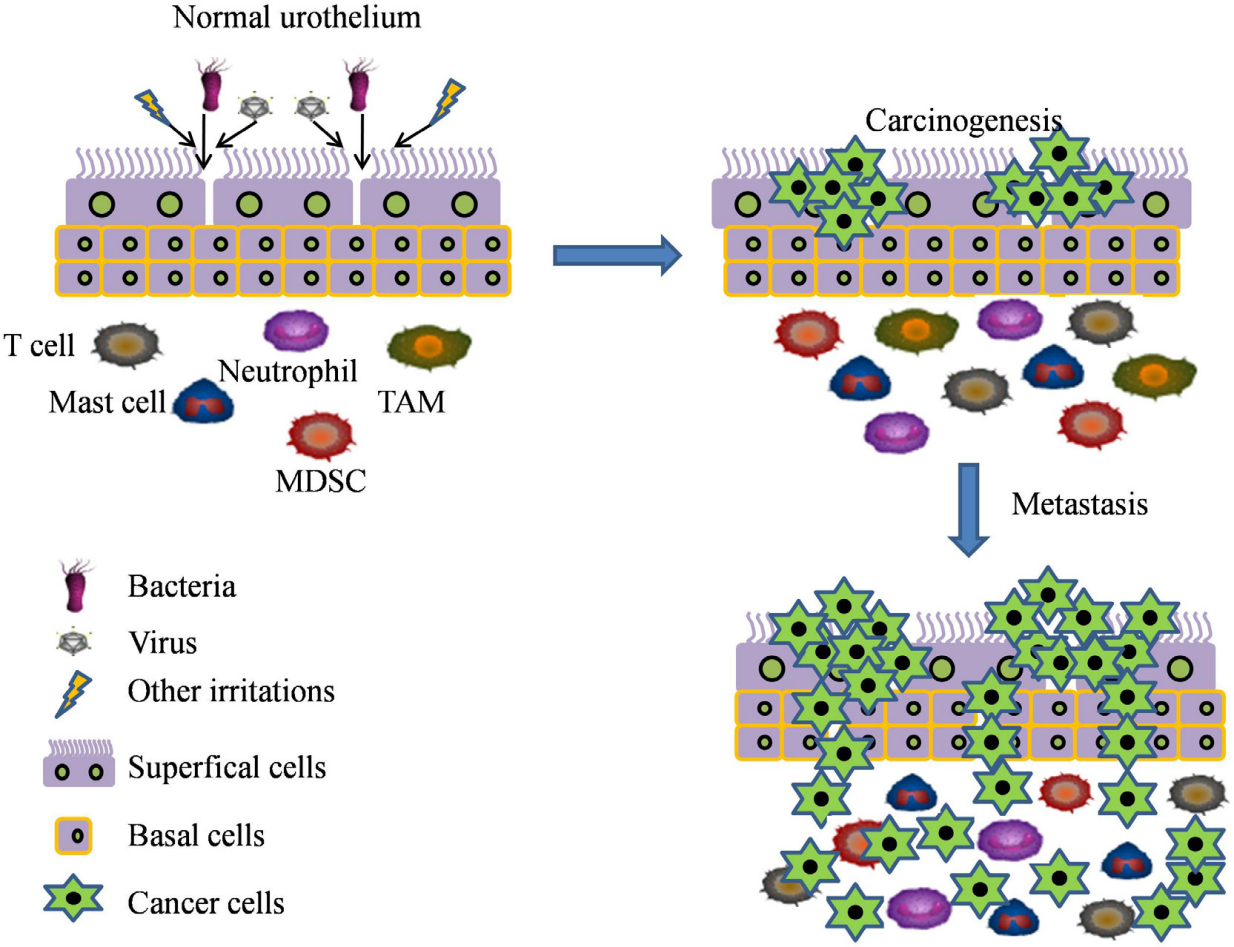
The inflammatory spectrum underlying the carcinogenesis and progression of bladder cancer Many factors such as infections (bacterial, *S. haematobium*, viral), proinflammatory cells (such as TAM, MDSC, T cells, mast cells and neutrophils), and chronic chemical or mechanical irritation are considered as a major risk factors of chronic inflammation. These factors can activate the inflammatory responses which contribute to the formation and development of bladder cancer.

The chronic inflammatory microenvironment in solid cancers is characterized by the presence of proinflammatory cells (such as macrophages, myeloid-derived suppressor cells, regulatory T cells, dendritic cells, mast cells, neutrophils and lymphocytes) and cytokines (such as tumor necrosis factor-α and interleukins) both in the supporting stroma and in tumor areas (Table 1). These chronic inflammatory cells and cytokines contribute to BCa formation and progression via multiple mechanisms (Figure 1).

**Table 1:** Inflammatory cells and cytokines in tumor microenvironment of bladder cancer

Class	Target	Biomarkers	Role in BCa	Histologic subtypes of BCa	Control	N	p value	References
**Inflammatory cells**								
TAMs	M1: TNF-α, IL	IL-8	Inhibition	TCC	Patients without treatment	12	p<0.05	52
	M2: TGF-β, IL	CD163	Promotion	TCC	Patients without treatment	99	p<0.05	54
MDSCs	PBMCs, IFN-γ	CD14(+)HLA-DR(-/low)	Promotion	TCC	Healthy human	64	p<0.01	64
MCs	c-Kit	c-Kit	Promotion	TCC	Normal bladder mucosa	78	p<0.05	82
	Stem cell	ALDH1	Inhibition	TCC	Healthy human	52	p<0.05	84
NLR	Unknown	NLR	Inhibition	TCC	NLR<2.7	899	p<0.05	88
**Inflammatory cytokines**								
TNF-α	MMP-9	MMP-9	Promotion	TCC	Without TNF-α	Cell lines	p<0.05	94
ILs	Unknown	IL-1α	Inhibition	Main TCC	Low IL-1α expression	164	p<0.05	95

ALDH1, aldehyde dehydrogenase 1 A1; PBMCs, peripheral blood mononuclear cells; TCC, Transitional cell carcinoma; SCC, squamous cell carcinoma.

### Macrophages

Tumor-associated macrophages (TAMs) play a dual role in BCa, depending on the different polarization states classified as M1 and M2 [50]. M1 macrophages can be induced by tumor necrosis factor (TNF)-α, interferon-γ (IF-γ), interleukin (IL)-1β, IL-6, IL-23, lipopolysaccharide (LPS), and show an inhibitory action in the initiation and/or progression of BCa (Table 1) [51, 52]. M2 macrophages are mainly activated by IL-4, IL-10, IL-13 or transforming growth factor-β (TGF-β) and associated with promotion of cancer cell proliferation, migration, invasion, metastasis and suppression of anti-tumor immune responses (Table 1) [53, 54].

Direct evidence for the role of TAM in the formation and development of BCa has recently been reported. OK-432, a streptococcus-derived anticancer immunotherapeutic agent, was demonstrated to suppress cell proliferation and metastasis through inducing M1 to secrete cytokines in BCa [55]. ATP-binding cassette transporter G1 (ABCG1) inhibited BCa growth through a phenotypic shift from a tumor-promoting M2 to a tumor-fighting M1 [56]. The predominance of M2-polarized macrophages in the stroma of low-hypoxic BCa was associated with Bacillus Calmette-Guérin (BCG) immunotherapy failure, possibly owing to immunosuppressive function of M2 [54]. Taken together, TAMs have been shown to be associated with carcinogenesis and progression of bladder, however, the molecular mechanisms that TAMs establish their tumor-inhibiting or tumor-promoting effect remain undefined.

### Myeloid-derived suppressor cells

Myeloid-derived suppressor cells (MDSCs) originate from bone marrow or from peripheral lymphoid organs, and their presence is associated with disease progression and reduced survival in many types of tumors [57, 58]. Accumulating evidence shows that induction of MDSCs is an important immune-evading strategy for cancer cells, which is linked to their immunosuppressive activity and the capacity to impair T cell function [59, 60]. MDSCs suppress anti-tumor immunity through multiple mechanisms, including a high level of arginase or tryptophan activity as well as nitric oxide (NO), reactive oxygen species (ROSs) and prostaglandin E(2) (PGE(2)) induction [59, 61].

BCa is a highly immunogenic malignancy and MDSCs are demonstrated to be critical mediators of BCa cell-associated immune suppression. Bladder cancer tissues spontaneously produce MDSCs-attracting CXCL8 (IL-8) and CCL22, which are correlated with poor prognosis of BCa [62]. The increased tumor infiltration of MDSCs with concomitant decrease of T cells and NK cells are shown in tyrosine kinase Rip2-deficient mice model of BCa, resulting in an enhanced incidence of metastases in BCa [63]. CD14(+)HLA-DR(-/low) cells, as a new subpopulation of MDSCs, display strong T-cell suppressive activity, and are associated with gender, tumor size, number of tumors and disease progression in the patients with BCa (Table 1) [64]. Thus, MDSCs may be a potential target for the tumorigenesis, progression and treatment of BCa.

### Regulatory T cells (Tregs)

Regulatory T cells (Tregs) play an essential role in the pathogenesis of inflammation and various autoimmune diseases, including cancer. Currently, numerous studies support the idea that Tregs can promote cancer progression by suppressing antitumor immune responses or expressing inflammatory cytokines [65, 66]. In bladder cancer, S1PR1 signaling in T cells can drive Treg accumulation in tumors through JAK/STAT3 activation, resulting in promoting BCa growth [67]. Moreover, the patients with bladder carcinoma show a relative enrichment of Tregs in peripheral blood compared with healthy controls [68, 69], and the suppression of Tregs contributes to an antitumor effect in an orthotopic BCa model [70]. However, the role of Tregs in chronically inflammatory environment of bladder cancer cells has not been explored.

### Dendritic cells

Dendritic cells (DCs) are professional antigen-presenting cells, thus, they play a crucial role in both the induction of antigen-specific immunity and the maintenance of tolerance [71]. The impairment of myeloid DC (mDC) counts and monocyte-derived DC (MoDC) function are closely associated with proliferation of superficial transitional cell carcinoma of the bladder (STCCB) [72]. Tumor-infiltrating dendritic cells (TIDCs) exhibit an abnormal phenotype and impaired function to stimulate T cells [73, 74]. Numerous studies have suggested that TIDCs contribute to tumor escape from immune surveillance by suppressing antitumor immune responses, therefore promoting tumor development [75, 76]. The high level of CD83(+) mature TIDCs is associated with a increased risk of muscle-invasive BCa [77]. In contrast, Xiang *et al*. reported that TIDCs were inversely correlated with the degree of malignancy and prognosis of bladder transitional cell carcinoma (BTCC) and the decrease in the number of TIDCs could have important relation to tumor immune evasion and immune tolerance [78]. Thus, TIDCs may be risk factors for BCa.

### Mast cells

Mast cells (MCs) are among potent proinflammatory cells that have been known to play an important role in variety of inflammation-associated diseases including cancer [79]. Several clinical studies suggested that MCs could influence the neoplasia and progression of BCa. The number of MCs within and around the tumor may be a useful prognostic indicator in patients with bladder carcinomas and MCs density is significantly higher in high-grade BTCC than low-grade BTCC [80, 81]. c-Kit positive MCs may contribute to tumor angiogenesis and play an important role in tumor invasion of the urinary bladder (Table 1) [82]. Recruited MCs in the tumor microenvironment are demonstrated to enhance bladder cancer metastasis through modulation of ERβ/CCL2/CCR2 EMT/MMP9 signals [83]. Whereas, stem cell marker-positive MCs are reduced in stroma of benign-appearing mucosa of BCa patients, indicating that MCs could be also involved in suppression of carcinogenesis (Table 1) [84]. However, the mechanisms of how mast cells influence the formation and progression of BCa are still unclear.

### Neutrophil-to-lymphocyte ratio

The neutrophil-to-lymphocyte ratio (NLR) in peripheral blood samples has been indicated as a crucial indicator for the systemic inflammatory response [85] and the prognosis of some solid malignancies including BCa [11, 86, 87]. Elevated serum NLR level among BCa patients undergoing radical cystectomy (RC) is associated with significantly increased risk for disease recurrence and progression (Table 1) [88, 89]. NLR is also associated with pathological response (pathR) in muscle-invasive bladder cancer (MIBC) patients who receive neoadjuvant chemotherapy (NC) [90]. Although the large number of clinical evidence has linked NLR with BCa progression, the molecular events by which NLR promotes BCa development and tumor recurrence are not fully understood.

### TNF-α

TNF-α is a key event for infectious disease and malignancy. The released TNF-α during inflammation is associated with the transformation of BCa due to induction of H_2_O_2_ [91]. The serum level of TNF-α is remarkably elevated in BCa patients with or without *schistosomiasis* infection, moreover, higher level of TNF-α is observed in T3 and T4 advanced-stage patients than T1 and T2 early-stage patients, indicating TNF-α level might contribute to the progression of BCa [92]. TNF-α gene promoter-308 A/G single nucleotide polymorphisms are recently found to be significantly associated with the tumor-invasive stage of BCa [93]. TNF-α is also implicated in promoting invasion and migration of BCa cells through stimulating the secretion of matrix metalloproteinases-9 (MMP-9) in the tumor microenvironment (Table 1) [94]. Taken together, TNF-α as a proinflammatory cytokine contributes to the formation and development of BCa.

### Interleukins

As proinflammatory cytokines, interleukins (ILs) have been involved in cancer initiation and progression. Low levels of IL-1α mRNA expression are associated with an increased risk for BCa-specific death (Table 1) [95]. IL-6, a major trigger of the signal transducers and activators of transcription 3 (STAT3) signaling pathway, have been implicated in regulation of tumor growth and metastasis of BCa. IL-6 level is positively linked with angiogenesis and the clinical outcome of BCa [96]. Interestingly, there is a conflicting report regarding the potential role of IL-6. Tsui and colleagues found that IL-6 attenuated tumorigenesis and cell invasion in human bladder carcinoma cells [97]. IL-8 over-production is an important factor in monomethylarsonous acid [MMA(III)]-induced malignant transformation of urothelial cells [98]. Increased expression of IL-8 is also correlated with tumor recurrence and poor prognosis of BCa [99]. The same as IL-6, IL-17 has a dual role in BCa. IL-17 can promote tumor growth through an IL-6-Stat3 signaling pathway [100]. In contrast, Baharlou *et al*. reported that reduced IL-17 levels in peripheral blood could be used as indicators for worse prognosis of BCa patients [101]. Serum IL-18 levels were significantly higher in BCa patients when compared to the control subjects, however, the relationship between IL-18 and tumor progression need to be further determined [102]. Therefore, interleukins may promote or inhibit bladder carcinogenesis.

## MOLECULAR BASIS OF CHRONIC INFLAMMATION IN INITIATION AND PROGRESSION OF BLADDER CANCER

**Figure 2:**
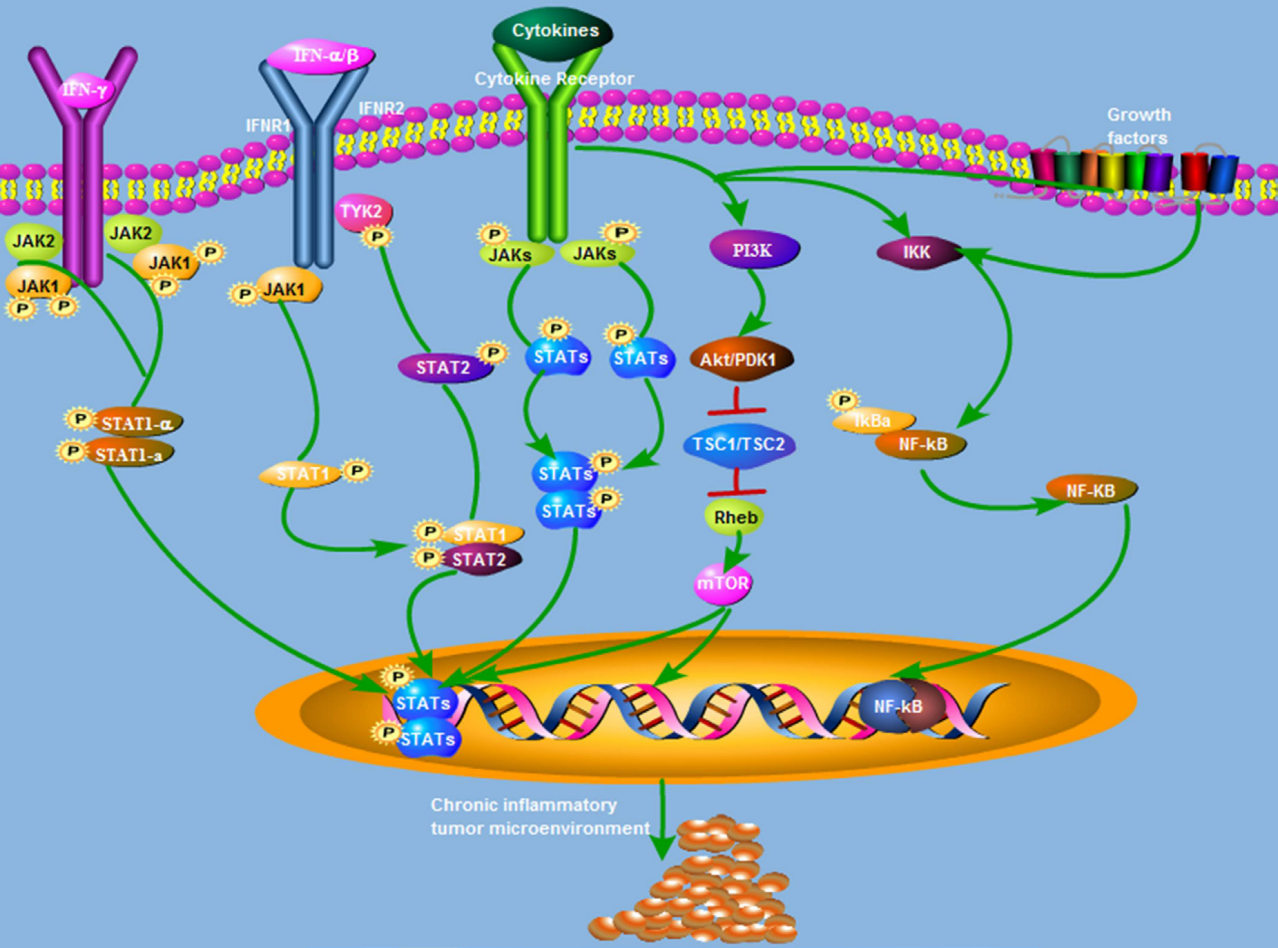
Summary of the signaling pathways underlying inflammatory response-mediated bladder cancer oncogenesis and progression

Several signaling pathways are known to be involved in the initiation and progression of BCa during inflammation, including Cyclooxygenase-2 (COX-2)/nitric oxide synthase (NOS), janus activated kinase (JAK)-STAT3, the nuclear factor-kappaB (NF-*κ*B), and phosphoinositide-3 kinase (PI3K)-Akt-mammalian target of rapamycin (mTOR) (Figure 2).

## CYCLOOXYGENASE-2/NITRIC OXIDE SYNTHASE (NOS)

Cyclooxygenase-2 (COX-2), the enzyme that converts arachidonic acid to prostaglandin H2, is stimulated by a number of inflammatory cytokines and plays a key role in tumorigenesis and cancer progression. COX-2 is commonly expressed in BCa cells but not in normal urothelium [103]. Nuclear localization of COX-2 is significantly associated with inflammation-mediated stem cell proliferation/differentiation in bladder tumorigenesis [25]. The overexpression of COX-2 is involved in the development of squamous cell carcinoma of the urinary bladder [104]. The high COX-2 expression is significantly associated with advancing grade and T stage of STCCB [105]. Although numerous studies suggest that COX-2 promotes bladder tumorigenesis and progression, its precise role remains non-conclusive. The data from Tadin *et al*. showed an inverse correlation exists between COX-2 expression and recurrence of non-muscle invasive bladder cancer (NMIBC) [106]. These results provide a pivotal role of COX-2 in the initiation and progression of BCa.

Nitric oxide (NO), generated by nitric oxide synthase (NOS), participates in the physiologic regulation of many diseases including cancer [107]. There are three NOS isoforms, endothelial (eNOS), neuronal (nNOS) and inducible (iNOS) [108]. NOS plays a role in simulating the pattern of fetal urothelium, which may be viewed as an oncofetal characteristic of this type of tumor [109]. Endogenously formed NO promotes cell proliferation in BCa cell lines [110]. NO generation from iNOS in the malignant epithelium and from eNOS in tumor stroma have an important potential in the angiogenesis of BCa [111]. However, the precise roles of NO and NOS of the inflammatory microenvironment in BCa carcinogenesis and progression remain to be defined.

### JAK-STAT3

JAK-STAT3 is a crucial signal pathway for the pathogenesis and progression of various inflammatory diseases including cancer. JAKs are key participants in signaling networks fueled by a variety of cytokine and growth factor receptors in the tumor microenvironment, including IL-6, IL-11, IL-27, interferon (IFN-α/β/γ) oncostatin M (OSM), leukemia inhibitory factor (LIF), epidermal growth factor (EGF) and others [112]. There are four JAK family members in mammalian cells, JAK1, JAK2, JAK3, and TYK2. JAKs mediate intracellular signaling cascades principally through creating STAT docking sites via phosphorylation of tyrosine residue [113]. Once tyrosine is phosphorylated, STAT proteins are rapidly transported from the cytoplasm to the nucleus and bind to DNA elements to elicit transcriptional outputs in a specific cell or tissue [114]. STAT3 is a member of STAT family and its phosphorylation at Tyr705 or Ser727 is widely mediated in a variety of cellular contexts, especially JAK2 [115].

JAK-STAT3 pathway is also involved in chronic inflammation-mediated malignant transformation of urothelial cells and the progression of BCa. Stat3 activation contributes to bladder cancer cell growth and survival [116, 117]. By contrast, the silencing of STAT3 significantly suppresses proliferation of T24 BC cells both *in vitro* and *in vivo* [118]. Therefore, inhibition of JAK-STAT3 signaling pathway provides us a potential therapeutic approach for BCa. Along the same lines, the activation of JAK-STAT3 pathway in tumor inflammatory microenvironment participates in the invasion and migration of BCa. Stat3 activation in urothelial stem cells may lead to direct progression of urothelial progenitor cells to carcinoma *in situ* (CIS) formation and subsequent MIBC [119]. CXCR4-mediated Stat3 activation promotes CXCL12-induced cell invasion in BCa [120]. STAT3 is also phosphorylated by interacting with c-JUN, which is necessary for migration and invasion activity of T24 BCa cells [121]. Cytoplasmic p27 activates STAT3 to induce a TWIST1-dependent epithelial-mesenchymal transition (EMT), resulting in increasing the invasion and metastasis of BCa [122]. Taken together, these reports have established a relationship between JAK-STAT3 pathway and inflammation-mediated bladder cancer.

### NF-κB

NF-*κ*B is a family of ubiquitously expressed transcription factors that are widely activated by various proinflammatory stimuli in the tumor microenvironment, including TNF-α, IL-1β and the IκB kinase (IKK) complex [115]. There are two major signaling pathways that mediate NF-κB activation: the canonical and noncanonical pathways. The canonical pathway (also known as classical pathway) depends on the IKK complex and is mainly activated by proinflammatory cytokines (TNF-α or IL-1β), growth factors as well as pathogenassociated molecular patterns (PAMPs), thereby enhancing cell survival and proliferation [123]. The non-canonical pathway (also known as alternative pathway) does not require the trimeric IKK complex and depends on the inducible processing of p100, a molecule functioning as both the precursor of p52 and a RelB-specific inhibitor [124].

NF-*κ*B activation has been reported in various human neoplasms including BCa. Fisetin, a dietary flavonoid, significantly reduces the incidence of N-methyl-N-nitrosourea (MNU) -induced bladder tumors by suppressing NF-*κ*B activation [125]. The curcumin potentiates the antitumor effect of Bacillus Calmette-Guerin (BCG) through the induction of TRAIL receptors and inhibition of NF-*κ*B in bladder cancer cells [126]. Nuclear expression of NF-*κ*B is correlated with histologic grade and T category in bladder urothelial carcinoma (UC) [127]. AKT-mediated NF-*κ*B activation upregulates snail expression and induces EMT, therefore, promoting tumor progression and metastasis in BCa [128]. NF-*κ*B activation also mediates angiogenesis and metastasis of BTCC through the regulation of IL-8 [129]. In addition, down-regulation of NF-*κ*B activation results in enhanced sensitivity of bladder cancer cells towards chemotherapeutic agents [130, 131].

### PI3K-Akt-mTOR

PI3K activation is initiated in response to cell surface tyrosine kinase receptor-ligand binding, and leads to the conversion of phophatidylinositol-4,5-bisphosphate (PIP2) to phosphatidylinositol-3,4,5-trisphosphate (PIP3) [132]. Subsequently, PIP3 recruits AKT and phosphoinositide-dependent kinase 1 (PDK1) to the plasma membrane, resulting in the phosphorylation of AKT either by PDK1 at Thr308 or by mTORC2 at Ser473 [133]. And then, AKT phosphorylation disrupts the interaction between tuberous sclerosis protein complex 1 (TSC1) and TSC2, and further inhibits the activation of Rheb that is a suppressor of mTOR function [134]. There are two functionally distinct mTOR complexes, mTORC1 and mTORC2. mTORC1 is mainly regulates protein translation and cell metabolism through its various downstream effectors. In contrast, mTORC2 is involved in actin cytoskeleton organization and cell survival. In addition, mTORC2 also regulates AKT activity by a feed-back loop [135].

PI3K-AKT-mTOR signaling pathway is frequently changed in several malignancies including BCa. Chen *et al*. evaluated 231 single-nucleotide polymorphisms (SNPs) in 19 genes in the PI3K-AKT-mTOR signaling pathway and they found four SNPs in raptor that were significantly associated with increased risk of BCa [136]. Moreover, activation of the PI3K-AKT-mTOR pathway was demonstrated to be correlated with tumor progression and poor survival of BCa patients [137]. Nicotine could induce acquired chemoresistance and increase tumor growth through activation of the PI3K-AKT-mTOR pathway in BCa [138]. Angiogenin (ANG), a member of RNase A superfamily, is recently demonstrated to promote tumor angiogenesis, tumorigenesis and metastasis of BCa by activating key downstream target molecules of PI3K-AKT-mTOR signaling pathway [139]. Although significant progress has been made in defining the role of this pathway in BCa, the mechanisms by which PI3K-AKT-mTOR pathway promotes BCa carcinogenesis and progression are not well known.

### MicroRNAs

MicroRNAs (miRNAs) are small noncoding molecules that regulate gene expression by silencing mRNA targets. miRNA dysregulation exhibits great regulatory potential during organismal development, cell proliferation and death, immunity, and inflammation [140]. Recently, increasing studies have linked miRNAs to inflammation during bladder cancer initiation and development.

TNF-α-related apoptosis-inducing ligand (TRAIL) shows a strong apoptosis-inducing effect on a variety of cancer cells including BCa. MiRNA-221 silencing promoted cell apoptosis induced by TRAIL in T24 cells [141]. The problem that adenoviral vector lacks the ability to discriminate cancer and normal cells seriously hurdles the clinical application of TRAIL therapy. To solve the problem, Zhao *et al*. applied miRNA response elements (MREs) of miR-1, miR-133 and miR-218 to confer TRAIL expression with specificity to bladder cancer cells. They found that miRNA response elements-based TRAIL delivery showed specific survival-suppressing activity on bladder cancer [142]. Notwithstanding a long list of miRNAs deregulated in BCa, there is very little overlap in the patterns of miRNA expression between inflammation and BCa. In order to evaluate the differential effects of inflammation on the bladder cancer, microRNA research may be an exciting and challenging field in the future.

## IMPLICATIONS FOR PREVENTION AND TREATMENT OF BLADDER CANCER

### Nonsteroidal anti-inflammatory agents and COX-2 inhibitors

Experimental and epidemiologic evidence strongly suggests that nonsteroidal anti-inflammatory drugs (NSAIDs) and COX-2 inhibitors have the potential as chemopreventive agents for cancer (Table 2) [143, 144]. NSAIDs (such as naproxen, sulindac, and their NO derivatives) show good preventive effects in a chemically induced urinary bladder cancer model [145]. As a NSAID, meloxicam treatment inhibits the development of bladder neoplastic lesions induced by BBN [146]. However, in a comprehensive meta-analysis, Zhang *et al*. evaluated the association between NSAIDs and BCa risk and they reported there was no significant association between use of aspirin or non-aspirin NSAIDs and BCa risk. However, non-aspirin NSAIDs use among long-term quitters might be associated with a decreased risk of BCa [147]. Celecoxib, a cyclooxygenase-2 (COX-2) inhibitor, promotes a striking inhibitory effect on BCa development, reinforcing the potential role of chemopreventive strategies based on COX-2 inhibition [148]. In contrast, the results from Sabichi *et al*. do not show a clinical benefit for celecoxib in preventing NMIBC recurrence [149]. So, whether the treatment of NSAIDs or COX-2 inhibitors may reduce the risk of BCa remains unclear.

**Table 2:** Active clinical drugs for the prevention and treatment of bladder cancer

Drugs	Identifier	Targets	Role	References
Meloxicam	NSAID	Unknown	Inhibition	146
Celecoxib	COX-2 inhibitor	COX-2	Inhibition	148
BCG	Vaccine	Cytokines/chemokines and T cells	Inhibition	151-155
IL-15	Gene therapy	T lymphocytes	Inhibition	161
IL-10 blocking antibodies	antibody	Th1	Inhibition	162
Ipilimumab	CTLA-4 inhibitor	CTLA-4	Investigation	164
Atezolizumab	PD-L1 inhibitor	PD-L1	Inhibition	167
Nivolumab	PD-1 inhibitor	PD-1	Inhibition	167
Pembrolizumab	PD-1 inhibitor	PD-1	Inhibition	164, 168

### BCG immunotherapy

BCG, a live attenuated form of *Mycobacterium bovis*, has been used to treat high grade NMIBC for almost 40 years, to reduce the risk of recurrence and progression (Table 2) [150]. Currently, several meta-analyses have confirmed that intravesical BCG is a first-line choice for reducing tumor recurrence and delaying or preventing progression to MIBC [151–153]. In addition, some clinical and experimental research also show that intravesical BCG is safe and effective in immunologically compromised patients with BCa [154], and BCG treatment suppresses the tumorigenesis and progression in a BBN-treated rodent model [155]. Although the precise mechanism of BCG antitumor response remains undefined, the current research suggest that it stimulates both an inflammatory tumor response as well as an immune response to kill the bladder cancer cells [156]. BCG presents its antitumor potential by secretion of cytokines/chemokines (such as IL-6, IL-8, CXCL1 and CXCR4) into tumor microenviroment, moreover, increased levels of cytokines/chemokines in serum are associated with enhanced survival in the animal model of BCa [157]. BCG's anti-tumor effects can be also attributed to the elimination of BCG-infected cancer cells by presentation of cancer cell antigens to immune cells, including natural killer (NK) cells, CD4(+) T cells and CD8(+) T cells [158, 159].

### ILs agonists or their antagonists

Based on the different role of ILs in the formation and development of BCa, ILs agonists or their antagonists are demonstrated to display distinct antitumor effect (Table 2). Intravesical IL-12 immunotherapy can induce tumor-specific systemic immunity against murine BCa [160]. IL-15 gene therapy inhibits cell survival in an orthotopic BCa model through inducing tumor-specific cytotoxic T lymphocytes [161]. IL-10 blocking antibodies enhances BCG induced Th 1 immune responses and anti-bladder cancer immunity [162]. In addition, there are 18 phase I/II cancer clinical trials (http://clinicaltrials.gov/) is assessing the efficacy of IL with or without conventional chemotherapeutics.

### Immune checkpoint inhibitors

Cytotoxic T-lymphocyte antigen 4 (CTLA-4) is a CD28 family member and is translocated to the membrane following activation of both CD4 and CD8 T cells. When expressed, CTLA-4 binds co-stimulatory B7 molecules with greater affinity than CD28 and generates a negative feedback loop to the early T cell response [163]. CTLA-4 targeting antibodies boost anti-tumor immunity through blocking the interaction between CTLA-4 and B7 ligands. Ipilimumab was the first FDA-approved CTLA-4 inhibitor for treatment of unresectable or metastatic melanoma as a monotherapy. Currently, one active trial (NCT01524991) is investigating the efficacy of the combination of Ipilimumab with conventional chemotherapeutics (Gemcitabine plus Cisplatin) as first-line treatment for patients with metastatic urothelial carcinoma (Table 2) [164]. The information about the antitumor activity of this combination is underway.

Programed cell death protein 1 (PD-1or CD279) is another coinhibitory receptor expressed on the surface of many different subtypes of tumor infiltrating leukocytes [165]. PD-1 is mainly activated by interacting with its ligands PD-L1 which is mainly expressed on dendritic cells, IFN-γ-treated monocytes and many types of cancer cells [166]. The first FDA-approved PD-L1 inhibitor in bladder cancer was atezolizumab, in May 2016, for patients with locally advanced or metastatic urothelial carcinoma after disease progression on or within 12 months of receiving platinum-based chemotherapy either before (neoadjuvant) or after (adjuvant) surgical treatment. This was followed by the approval of PD-1 blockade drug nivolumab in February 2017 for treatment of locally advanced or metastatic urothelial carcinoma whose disease has progressed during a period of up to 1 year after first-line platinum-containing chemotherapy [167]. Another PD-L1 inhibitor pembrolizumab showed a 24.1% overall response rate for urothelial cancer patients. To assess the efficacy of the combination of pembrolizumab with conventional chemotherapeutics, several clinical trials (NCT02335424, NCT02351739 and NCT022456436) have been launched [164, 168]. As mentioned, PD-1 or PD-L1 inhibitors have shown promising roles in the treatment of BCa patients (Table 2). However, future research is necessary to characterize therapeutic response and identify predictive markers of response for these drugs.

## CONCLUSIONS AND PERSPECTIVES

Chronic inflammation is an important risk factor for the development of urinary bladder cancer. Many factors are involved in inflammation-associated cancer risk, including infections (bacterial, *S. haematobium*, viral), immunological disorders, and chronic chemical and mechanical irritation. Multiple proinflammatory molecules and signaling pathways in tumor microenvironment may elicit a crucial role in formation and progression of BCa. Therefore, several cytokine/chemokine antagonists or their antagonists and some signaling pathway inhibitors as potential agents of chemoprevention and treatment against BCa are in clinical investigation.

However, several gaps still exist in our knowledge that should be addressed in the future. Firstly, the question whether we should try to inhibit local inflammatory reactions or systemic inflammation responses is paradoxical since neoplastic disorders are usually associated with a local but not a systemic inflammatory response, which should be considered in the ongoing clinical trials where systemic anti-inflammation agents are used in the prevention and treatment of BCa. Second, it is unclear whether inflammation is sufficient for the formation of cancer, which means whether inflammation can induce carcinogenesis in the absence of an exogenous carcinogenic agent. Third, it has been known that the outcomes of inflammation activation are highly dependent on the treatment characteristic and tumor types. Moreover, the signaling pathways involved in chronic inflammatory tumor microenvironment and their possible crosstalk among themselves are complicated. So, what is needed to be better known include elucidating the specific impact of the chronic inflammation on bladder carcinogenesis and progression, and determining the exact molecular mechanisms by which the chronic inflammation can regulate the growth, survival, invasion and metastasis. However, our increased understanding of the role of chronic inflammation in tumor microenvironment will hopefully provide us an attractive therapeutic strategy to impede carcinogenesis, inhibit cancer cell survival and metastasis for bladder cancer patients.
